# Genomic and Phenotypic Evaluation of Safety, Probiotic Potential, and Aroma Production of *Saccharomyces cerevisiae* FOSU-QQT

**DOI:** 10.3390/molecules31132310

**Published:** 2026-07-01

**Authors:** Shao-Fu Feng, Hui-Lan Tan, Qi-Qing Tan, Xin-An Zeng, Lang-Hong Wang, Yan-Yan Huang, Man-Sheng Wang

**Affiliations:** 1 Guangdong Provincial Key Laboratory of Intelligent Food Manufacturing, Foshan University, Foshan 528225, China; t2120295904@126.com (S.-F.F.); 13717484371@163.com (H.-L.T.); 18607587770@163.com (Q.-Q.T.); xazeng@scut.edu.cn (X.-A.Z.); wlhong@fosu.edu.cn (L.-H.W.); 2 School of Food Sciences and Engineering, South China University of Technology, Guangzhou 510640, China; 3 Institute of Bast Fiber Crops, Chinese Academy of Agricultural Sciences, Changsha 410205, China

**Keywords:** *Saccharomyces cerevisiae*, whole-genome, phenotypic analysis, safety, probiotics, aroma-producing

## Abstract

*Saccharomyces cerevisiae* FOSU-QQT (SC.QQT), isolated from pineapple pomace wine, exhibits favorable aroma-producing capabilities. In this study, we performed integrated genomic and phenotypic analyses to comprehensively evaluate its safety profile, probiotic potential, and aroma-producing characteristics. Whole-genome sequencing (WGS) assembly predicted a genome size of 30,256,254 bp, encompassing 12,899 genes with a total coding length of 22,062,659 bp and an average GC content of 37.30%. Preliminary safety assessments, including hemolysis tests, antibiotic susceptibility profiling, and antibacterial activity assays, were complemented by in silico screening for antibiotic resistance-associated genes. Functional tolerance assays, specifically resistance to simulated gastrointestinal fluid, acid stress, and bile salts, demonstrated that SC.QQT exhibited robust survival under physiologically relevant gastrointestinal conditions. Collectively, these findings support its potential as a promising probiotic candidate with notable resilience, although further in vivo validation is required to confirm its application value. Additionally, gas chromatography–mass spectrometry (GC–MS) analysis of volatile compounds in pineapple pomace wine indicated that the presence of aroma-related genes in SC.QQT may enhance overall flavor complexity and intensify fruity aromatic notes during fermentation, underscoring its distinctive utility in fruit wine bioprocessing.

## 1. Introduction

Probiotics are live microorganisms that confer health benefits to human and animal hosts, particularly by supporting gastrointestinal function through the restoration and maintenance of a balanced gut microbiota. Given their role in promoting intestinal homeostasis, these microbes are commonly termed “beneficial” or “health-promoting” bacteria [1]. While most well-characterized probiotic microorganisms are bacteria such as *Bifidobacterium* and *Lactobacillus*, accumulating evidence from controlled studies indicates that select yeasts also possess probiotic properties [2].

*Saccharomyces cerevisiae* and *Saccharomyces boulardii* are currently the only commercially available probiotic yeast species approved for human consumption [3]. *S. boulardii* is widely recognized as the gold standard for yeast probiotics due to its proven clinical efficacy; however, exploring non-conventional *S. cerevisiae* strains with specific technological traits remains a significant area of interest [4]. Among them, *S. cerevisiae* is one of the most extensively employed yeasts in industrial biotechnology. It has a long-standing history of safe and effective application in food fermentation, including the fermentation of alcoholic beverages such as beer and wine, as well as in leavened bread manufacturing [5]. Regular intake of probiotic microorganisms has been associated with multiple health benefits, including the modulation of intestinal motility and barrier function, enhanced lactose digestion, immunomodulatory effects, and inhibition of enteric pathogens [6].

SC.QQT was isolated from pineapple pomace wine. Previous studies have demonstrated that this strain displays remarkable tolerance to low pH, high ethanol concentrations, and sulfur dioxide, which are key stressors encountered during fruit wine fermentation, along with a strong capacity for the biosynthesis of aroma compounds. This dual capability, combining robust stress tolerance with aroma production, distinguishes SC.QQT from many established probiotic yeasts and addresses the growing demand for multifunctional starter cultures in food biotechnology [7].

Evaluating the safety and probiotic properties of microbial strains is essential for their safe and effective application in human and animal health [8]. Probiotic yeasts, particularly those with demonstrated tolerance to gastric acidity (low pH) and intestinal bile salts, frequently exhibit potent antimicrobial activity against clinically relevant pathogens and food-spoilage microorganisms [9]. WGS permits comprehensive, genome-scale safety assessments, enabling the precise identification of potential virulence factors, antibiotic resistance genes, and metabolic pathways related to probiotic functionality [7,10]. Moreover, WGS enables comparative genomic analyses, which are essential for elucidating the genetic basis of strain-specific phenotypic traits, such as stress tolerance and aroma production. Comparative genomics provides a powerful framework for elucidating conserved as well as strain-specific genetic determinants of probiotic functionality, both within and across yeast species. Consequently, an integrative approach that combines rigorous phenotypic assays with deep genomic analysis is critical for robustly evaluating both the safety profile and probiotic potential of candidate strains [11]. In this study, we comprehensively evaluated the safety profile, probiotic properties, and aroma-producing capabilities of strain SC.QQT through WGS, comparative genomic analysis, and complementary phenotypic assays, thereby establishing a solid theoretical foundation for its future development and application.

## 2. Results and Discussion

### 2.1. Whole-Genome Sequencing and Functional Analysis of SC.QQT

**Figure 1:**
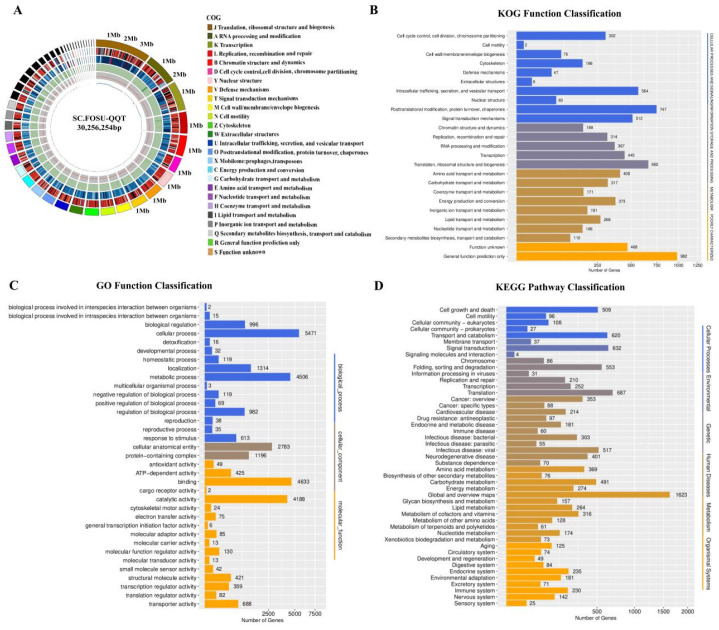
Genomic profile of SC.QQT and functional annotation in different databases. (**A**) shows the chromosome map of SC.QQT, (**B**) shows the KOG functional classification, (**C**) shows the GO functional classification, and (**D**) shows the KEGG functional classification. Sequencing was performed using DNBSEQ (76×) and PacBio (113×) platforms; genome assembly was accomplished using hifiasm (N50 = 941,290 bp); functional annotation was conducted using the KOG, GO, and KEGG databases. The BUSCO completeness assessment reached 97.95%. J: Translation, ribosomal structure and biogenesis; A: RNA processing and modification, K: Transcription; L: Replication, recombination and repair; B: Chromatin structure and dynamics; D: Replication, recombination and repair; Y: Nuclear structure; V: Defense mechanisms; T: Defense mechanisms; M: Cell wall/membrane/envelope biogenesis; N: Cell wall/membrane/envelope biogenesis; Z: Cytoskeleton; W: Extracellular structures; U: Intracellular trafficking, secretion, and vesicular transport; O: Posttranslational modification, protein turnover, chaperones; X: Mobilome: prophages and transposons; C: Energy production and conversion; G: Carbohydrate transport and metabolism; E: Amino acid transport and metabolism; F: Nucleotide transport and metabolism; H: Coenzyme transport and metabolism; I: Lipid transport and metabolism; P: Inorganic ion transport and metabolism; Q: Secondary metabolites biosynthesis, transport and catabolism; R: General function prediction only; and S: Function unknown.

Based on WGS analysis, the genome of strain SC.QQT has a total length of 22,062,659 bp, with a genome size of 30,256,254 bp and a GC content of 37.30%. Genome annotation identified a total of 12,899 protein-coding genes, accounting for 72.92% of the total genome length (Figure 1A). Non-coding RNA prediction revealed 703 tRNAs, 159 rRNAs (including 56 5S rRNAs, 50 18S rRNAs, and 53 28S rRNAs), 117 sRNAs, 222 snRNAs, and 411 miRNAs (Table 1). These observed genomic metrics reflect the use of the SuperGenome assembly strategy, which retains fragmented scaffolds and redundant sequences, resulting in an inflated genome size and gene count. Prior to assembly, K-mer analysis of this non-model, wild-type isolate predicted a genome size of approximately 32 Mb, which is in agreement with the final assembly size.

To further assess assembly redundancy, we examined multiple complementary metrics. K-mer analysis predicted a genome size of approximately 32 Mb, which is in close agreement with the final assembly size (30.26 Mb), indicating no substantial inflation. Repetitive sequences account for only 4.95% of the genome, with transposable elements representing 3.95%. The assembly comprises only 463 scaffolds, with an N50 of 941 kb and no gaps, reflecting high contiguity and limited fragmentation-driven redundancy. Moreover, collinearity analysis revealed no large-scale structural variations or segmental duplications compared to other *S. cerevisiae* strains. Although the exact BUSCO duplication percentage could not be retrieved, these metrics collectively suggest that the elevated gene count primarily arises from the SuperGenome assembly strategy rather than from genuine genomic duplications.

Regarding ploidy and assembly inflation, K-mer frequency analysis (15-mer and 21-mer) of the Illumina sequencing data revealed a single major depth peak with no secondary peak at half depth, confirming a haploid genome. This observation excludes the possibility that the inflated gene count results from the assembly of a diploid genome erroneously doubling gene numbers. The SuperGenome assembly strategy, which intentionally retains fragmented scaffolds and redundant sequences, directly contributes to the observed elevation in genome size and gene count. These metrics should be interpreted as assembly outputs rather than a biologically validated gene repertoire. Consequently, any downstream inference based on raw gene counts, unique gene numbers, or functional category abundances must be considered preliminary and hypothesis-generating and requires experimental validation.

The KOG (Eukaryotic Orthologous Groups) database classifies orthologous proteins from eukaryotic genomes based on sequence and evolutionary relationships. In the SC.QQT genome, 7065 genes (54.77%) were assigned KOG functional annotations (Figure 1B). The annotation results revealed that 2485 genes are involved in cellular processes and signaling, 1957 genes in information storage and processing, and 2004 genes in metabolism, while 1450 genes remained functionally uncharacterized. Among these, the category with the greatest number of associated genes was general function prediction only (982 genes), followed by posttranslational modification, protein turnover, and chaperones (747 genes), and translation, ribosomal structure and biogenesis (662 genes).

Based on functional annotation analysis using the Gene Ontology (GO) database, 9195 genes (71.28%) in the SC.QQT genome were assigned functional annotations. These annotated genes were classified into three main functional categories: the largest number of genes were involved in biological processes (14,330 genes); followed by genes related to molecular function (11,235 genes); whereas the number of genes related to cellular components was relatively small, totaling 3979. Notably, a single gene can be annotated with multiple GO terms (e.g., participating in both a biological process and a molecular function); therefore, the sum of gene counts across categories exceeds the total number of annotated genes, which is a standard feature of GO analysis. As indicated in the sequencing report, these annotations are descriptive and do not involve statistical enrichment testing. The distribution of functional annotations provides key theoretical and data support for in-depth analysis of the biological functions of the SC.QQT genome (Figure 1C).

The KEGG database enables systematic analysis of metabolic pathways and functional annotations of gene products within cells. In the SC.QQT genome, 7499 genes (58.13%) were assigned KEGG functional annotations (Figure 1D). Functional classification revealed that 1360 genes are involved in cellular processes, 673 in environmental information processing, 1819 in genetic information processing, 4006 in metabolism, and 1216 in organismal systems. Among these, the largest category was “Global and overview maps” (1623 genes), followed by “Translation” (687 genes) and “Signal transduction” (632 genes). Additionally, 369 genes associated with amino acid metabolism, 491 with carbohydrate metabolism, and 37 with membrane transport were annotated.

The detailed annotation data, including gene identifiers, scaffold assignments, genomic coordinates, predicted functions, and annotation confidence levels, are provided in Table S3.

It should be emphasized that all gene numbers, unique gene counts, and functional category abundances presented herein are assembly derived predictions and have not been experimentally validated. Consequently, they should be interpreted as preliminary and hypothesis-generating.

### 2.2. Comparative Genomic Analysis of SC.QQT and Other Five S. Cerevisiae

**Figure 2:**
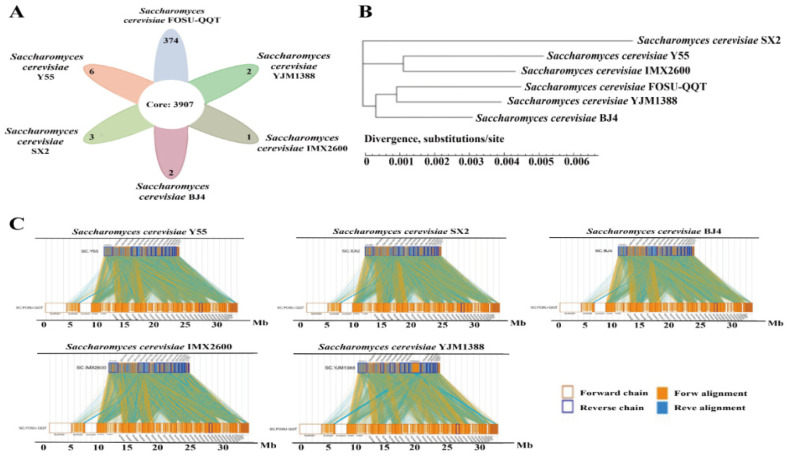
Comparative genomic analysis of SC.QQT and five other *S. cerevisiae* strains (SC.Y55, SC.SX2, SC.BJ4, SC.YJM1388, SC.IMX2600). (**A**) shows the core genes and unique genes of the six strains, (**B**) shows a Phylogenetic tree based on the sequence similarity of 3907 core genes, and (**C**) shows the collinearity analysis of SC.QQT and the five other *S. cerevisiae* strains at the nucleic acid level.

Comparative genomics enables the analysis of genome maps and sequences to investigate gene function, expression regulation, and evolutionary conservation. In the NCBI database, SC.QQT exhibited high genetic similarity to five other *S. cerevisiae* strains: SC.Y55, SC.SX2, SC.BJ4, SC.YJM1388, and SC.IMX2600. Core genome analysis of these six strains identified 3907 core genes, among which the assembly predicted 374 unique genes in SC.QQT (Figure 2A). A phylogenetic tree constructed based on the sequence similarity of the 3907 core genes revealed a closer genetic relationship between SC.QQT and SC.YJM1388 (Figure 2B), suggesting that these two strains may share similar physiological and biochemical characteristics.

Genomic collinearity analysis is used to identify structural variations between a sequenced genome and a reference genome. This approach enables simultaneous visualization of multiple mutation types and facilitates the localization of homologous conserved blocks and strain-specific regions [12]. It is worth noting that no significant large-scale structural variations (e.g., long-range indels or chromosomal inversions) were detected in the SC.QQT genome. Collinearity analysis revealed high structural conservation between SC.QQT and SC.YJM1388, whereas local inversions were observed in comparisons with SC.Y55, SC.SX2, and SC.IMX2600. These observations describe structural conservation without implying specific evolutionary mechanisms.

### 2.3. Safety Assessment of SC.QQT

#### 2.3.1. Hemolytic Analysis

**Figure 3:**
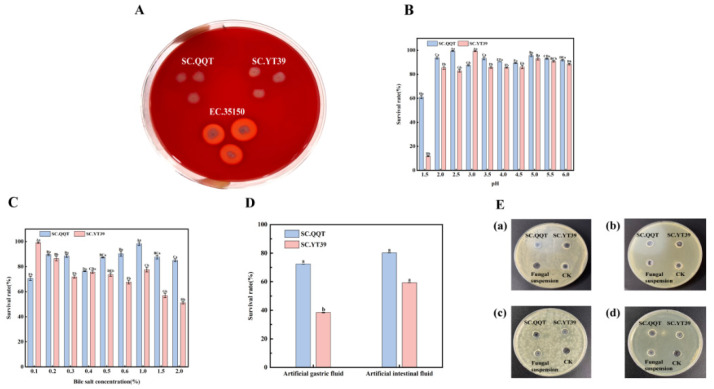
Safety Analysis and probiotic analysis of SC.QQT. (**A**) shows the results of the hemolysis test (28 °C, 48 h). (**B**) and (**C**) show the tolerance results of SC.QQT and SC.YT39 in YPD liquid medium containing acid (pH 1.5, 2.0, 2.5, 3.0, 3.5, 4.0, 4.5, 5.0, 5.5, and 6.0; 28 °C, 48 h) and bile salts (0.1%, 0.2%, 0.3%, 0.4%, 0.5%, 0.6%, 1.0%, 1.5%, and 2.0%; 28 °C, 48 h), respectively. (**D**) shows the survival of SC.QQT and SC.YT39 in simulated artificial gastric (pH 2.5, pepsin 0.3%) and intestinal fluids (pH 8.0, trypsin 0.1%). (**E**) shows the results of the antibacterial test ((**a**), *E. coli* ATCC 35150; (**b**), *Listeria monocytogenes* ATCC 19115; (**c**), *S. aureus* ATCC 25923; (**d**), *Salmonella enterica* ATCC 9120). Different uppercase letters indicate significant differences between groups, while different lowercase letters indicate significant differences between the experimental groups and the control group (*p <* 0.05). All experiments were performed in triplicate (*n* = 3), and the data are presented as the mean ± standard deviation (SD). SC.YT39 was used as a negative control.

The hemolysis of probiotic strains is one of the important criteria affecting their safety. According to the European Food Safety Authority (EFSA), evaluation of hemolytic activity is strongly recommended for probiotics intended for food use [13]. Hemolysis is typically caused by bacterial hemolysins, which induce erythrocyte lysis and may contribute to infections ranging from mild to severe [14]. As shown in Figure 3A, the positive control strain *E. coli* 35150 produced clear zones around colonies, indicating β-hemolysis. In contrast, neither SC.QQT nor SC.YT39 exhibited transparent zones, demonstrating γ-hemolysis (non-hemolytic).

Collectively, the absence of hemolytic activity and known virulence genes suggests a preliminary food-grade safety profile for SC.QQT. Furthermore, no hemolysis-associated genes—such as *LIP1* (encoding a phospholipase) or *FPS1* (involved in regulating substance uptake and metabolism)—were identified in the whole-genome sequence of SC.QQT [15,16]. However, comprehensive safety validation, including cytotoxicity assays and biogenic amine production tests, is recommended for future industrial applications.

#### 2.3.2. Analysis of Antibiotic Resistance

The excessive and inappropriate use of antibiotics has led to the increasingly prominent issue of antibiotic resistance. According to the FAO/WHO, even probiotic microorganisms that are generally recognized as safe (GRAS) must undergo antibiotic resistance assessments [17]. In this study, both SC.QQT and the control strain SC.YT39 exhibited resistance to 31 antibiotics, including penicillin and chloramphenicol (Table 2). However, this broad-spectrum resistance should not be overinterpreted as acquired resistance. In the context of yeast biology, this phenomenon is largely attributable to intrinsic characteristics of the strain rather than clinical multidrug resistance [18]. As a eukaryote, *S. cerevisiae* fundamentally lacks the specific molecular targets of most antibacterial agents (e.g., penicillin targets bacterial cell wall synthesis, whereas chloramphenicol targets prokaryotic ribosomes) [19]. Furthermore, the dense cell wall and membrane structure of yeast act as a natural permeability barrier, effectively blocking the intracellular entry of these compounds [18].

However, a more accurate assessment of antibiotic resistance genes in SC.QQT requires further prediction using whole-genome information. Comparative analysis of SC.QQT gene data against the CARD database revealed only one specific tetracycline resistance gene, *tet(W/N/W)*, with no other classes of antibiotic resistance genes annotated. *tet(W/N/W)* is a chimeric gene encoding a tetracycline resistance ribosomal protection protein, which confers resistance by protecting the ribosome at its binding site rather than by actively pumping the drug out of the cell [20]. Tetracyclines inhibit protein synthesis by preventing aminoacyl–tRNA binding to the ribosome. These drugs possess broad-spectrum antibacterial activity, and their widespread use has driven the emergence of resistance and its dissemination via horizontal gene transfer [21]. Studies have shown that the *tet(W/N/W)* gene can be transmitted among eukaryotic microorganisms through horizontal gene transfer, and strains harboring this gene typically exhibit resistance to tetracycline antibiotics [22]. Notably, *S. boulardii*—the most widely used probiotic yeast—exhibits variable antibiotic susceptibility, with some strains showing azole resistance but sensitivity to other classes [4]. In contrast, the resistance observed in SC.QQT is strictly intrinsic (lacking prokaryotic drug targets) and limited to a single tetracycline resistance gene (*tet(W/N/W)*), with no risk of plasmid-mediated transfer—consistent with EFSA guidelines for food-grade probiotics [13,17].

The absence of annotated antibiotic resistance genes in other categories may be attributed to alternative resistance mechanisms mediated by non-resistance genes, such as enzymatic inactivation or modification, enhanced efflux pump activity, and alterations in outer membrane permeability [23]. Moreover, complete genome sequencing confirmed the absence of plasmids in SC.QQT, suggesting a low risk of plasmid-mediated transfer of antibiotic resistance genes in this strain. In summary, the antibiotic resistance profile of SC.QQT is predominantly characterized by naturally intrinsic resistance. The lack of plasmids significantly minimizes the potential for horizontal transfer of the *tet(W/N/W)* gene, supporting the strain’s potential for safe application as a food-grade microorganism, pending further toxicological evaluation.

### 2.4. Probiotic Analysis of SC.QQT

#### 2.4.1. Analysis of Acid Tolerance Results

The human gastrointestinal tract presents a highly complex and variable environment, with pH being a key factor affecting yeast viability. Under normal conditions, gastric pH ranges from 1.0 to 4.0 [24]. Previous studies on probiotic *Saccharomyces* strains have reported survival rates of 50–90% after 2 h of exposure to pH 2.0–3.0 [25]. In the present study, SC.QQT achieved a maximum survival rate of 99.50% at pH 2.5, reflecting its adaptation to the low-pH environment of fermenting pineapple pomace wine. Across the pH range of 1.5–6.0, SC.QQT consistently exhibited higher survival rates than the control strain SC.YT39. Notably, after 48 h of cultivation at pH 1.5, the survival rate of SC.YT39 was significantly lower (11.86%), far below that of SC.QQT (Figure 3B). It should be noted that while these assays are standard for preliminary probiotic screening, they do not directly reflect in vivo host interactions or health benefits, which require dedicated validation.

In response to acidic environments, the SC.QQT genome assembly contains sequences predicted to encode a complete F0F1-ATPase system and a V-type ATPase system, which efficiently regulate intracellular pH through ATP consumption [26]. Additionally, two Na^+^/H^+^, two K^+^/H^+^, and five Ca^2+^/H^+^ antiporter genes are present, which also contribute to pH homeostasis by pumping H^+^ out of the cell at the expense of ATP. Although the presence of these genes correlates with the observed acid tolerance, direct functional validation is required to confirm their specific contributions to stress survival.

#### 2.4.2. Analysis of Bile Salt Tolerance Results

Bile salts are sodium or potassium derivatives of bile acids present in the intestine, where they participate in fat digestion and absorption [27]. Their presence in the gastrointestinal tract is a critical factor affecting microbial viability; therefore, tolerance to bile salts is an essential criterion for selecting potential probiotic microorganisms [28]. As the bile salt concentration increased gradually, SC.QQT consistently exhibited higher survival rates than the control strain SC.YT39, reaching a maximum of 98.34% at a concentration of 1.0%. In contrast, after 48 h of cultivation at 2.0% bile salts, the survival rate of SC.YT39 was significantly lower (51.25%), far below that of SC.QQT, indicating that SC.QQT possesses greater tolerance under high bile salt stress (Figure 3C).

Bile salt hydrolase (BSH) is a key intracellular enzyme produced by gut microbiota, catalyzing the hydrolysis of the amide bond in conjugated bile acids to generate free bile acids and release amino acids such as glycine and taurine, thereby influencing the enterohepatic circulation of bile acids [29]. BSH enhances bile salt tolerance by reducing local bile salt levels. However, no genes encoding bile salt hydrolases were identified in the SC.QQT genome. An alternative mechanism of bile salt tolerance involves the efflux of bile salts from cells via the efflux system of the bile salt transporter family (ABC transporter family) [30]. Collectively, these findings suggest that SC.QQT is well adapted to the bile salt challenge present in the human intestinal environment, although this adaptation requires confirmation through in vivo colonization studies.

#### 2.4.3. Analysis of Artificial Gastrointestinal Fluid Tolerance Results

Probiotic microorganisms, when administered in adequate amounts, confer health benefits to the host by improving the balance of the gut microbiota. To exert such benefits, probiotics must survive in sufficient numbers during gastrointestinal transit, tolerate acidic conditions, bile salts, and digestive enzymes, and subsequently adhere to and colonize the intestinal epithelium [31]. Following 3 h of exposure to simulated gastric juice, the survival rate of SC.QQT reached 72.39%, whereas that of the control strain SC.YT39 was 38.44%, indicating that SC.QQT exhibits strong tolerance to simulated gastric juice and remains largely unaffected by this treatment (Figure 3D).

Intestinal fluid—primarily duodenal fluid, which includes pancreatic juice, bile, and intestinal secretions—typically presents a mildly alkaline pH. For probiotics to function effectively in the gut, they must tolerate the trypsin and alkaline environment of the small intestine. Further experiments showed that after simulated intestinal fluid treatment, the survival rate of SC.QQT increased to 80.23%, while that of SC.YT39 rose to 59.35%. These results demonstrate that SC.QQT possesses robust in vitro tolerance to simulated gastrointestinal conditions compared with SC.YT39, a prerequisite for, but not direct evidence of, in vivo probiotic functionality (Figure 3D).

In response to adaptive osmotic stress challenges in the gastrointestinal tract, the SC.QQT genome assembly was predicted to encode a multicomponent ABC-family protein-dependent transport system (*opuABCD*), a complete F0F1-ATPase system, a V-type ATPase system, two Na^+^/H ^+^, two K/H^+^, and five Ca^2^/H^+^ countertransport genes, along with associated chaperone proteins and Clp proteases (ClpP, ClpB, ClpX) (Table S2). These components suggest a genomic capacity for regulating cellular responses to osmotic stress. Although the presence of these genes supports the observed robustness in simulated gastrointestinal fluids, definitive proof of their causal role awaits targeted gene knockout and complementation studies.

#### 2.4.4. Bacteriostasis Analysis

One of the most desirable properties of probiotic yeasts is their antibacterial activity against pathogens colonizing various mucosal surfaces [6]. In the present study, neither the stock solution of SC.QQT nor that of the control strain SC.YT39 produced visible inhibition zones against the four tested pathogenic bacteria (Figure 3E). The antagonistic activity of yeast isolates against pathogens is generally attributed to nutrient competition, organic acid production, high ethanol levels, and secretion of antimicrobial compounds such as lytic toxins or mycotoxins [32]. The absence of antimicrobial activity observed here is consistent with previous findings, in which potential probiotic yeasts isolated from cheese also exhibited no antimicrobial activity against *Escherichia coli* and *Staphylococcus aureus* [33]. Moreover, in-depth analysis of the SC.QQT whole-genome sequence revealed no genes associated with the biosynthesis pathways of bacteriocins or antimicrobial peptides, suggesting that this strain may exert its antibacterial effects through alternative mechanisms.

Adhesion-related genes, such as the cell adhesion complex protein (Bystin), were predicted in the SC.QQT assembly. Although co-aggregation with pathogens has been reported as a probiotic trait that may prevent pathogen colonization [32], this mechanism was not experimentally tested in the current study. The presence of these genes suggests a potential for such interactions, but their functional role requires validation via co-aggregation assays. Notably, co-aggregation could also exert potentially detrimental effects by facilitating pathogen attachment to human tissues [34]. Therefore, further research is needed to determine the specific effects of co-aggregation and the potential antimicrobial properties of yeast isolates, which are typically dependent on environmental conditions.

Importantly, the absence of inhibition zones aligns with the safety assessment of SC.QQT, as non-inhibitory yeasts are less likely to disrupt the host’s normal intestinal microbiota. Probiotics that produce inhibitory substances may suppress beneficial gut flora, whereas non-inhibitory *S*. *cerevisiae* strains are less likely to disturb intestinal microecological homeostasis [35]. Thus, the non-inhibitory characteristic of SC.QQT reduces potential disturbances to the intestinal microecological balance, further supporting its favorable safety profile.

### 2.5. Analysis of the Aroma Characteristics of SC.QQT

**Figure 4:**
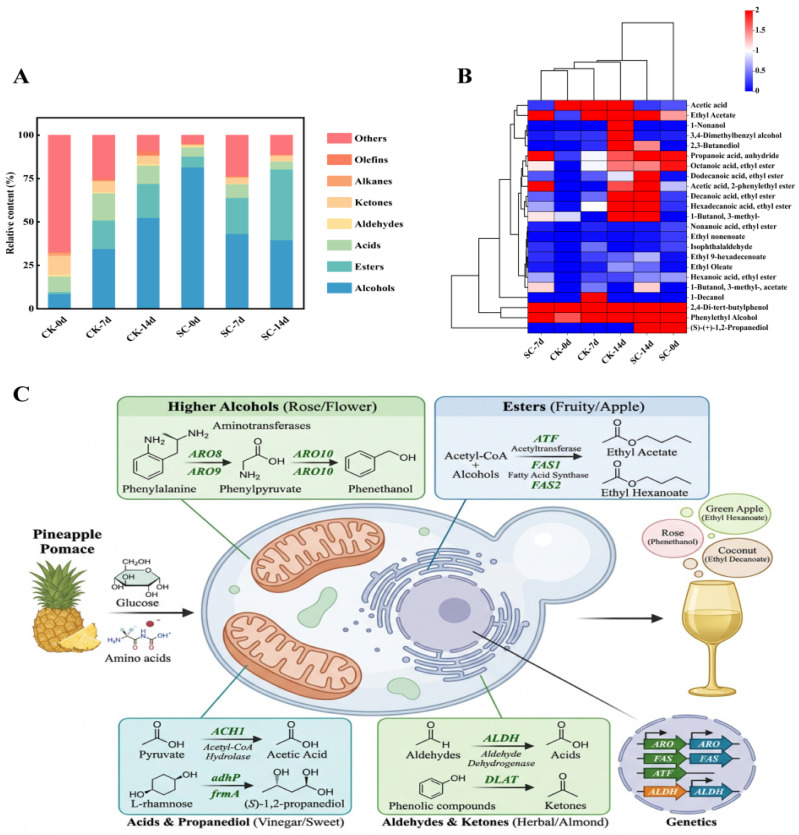
Analysis of the Aroma-Producing Characteristics of SC.QQT. (**A**) shows the accumulation diagram of various volatile substances across different treatment groups (all experiments were performed in triplicate (*n* = 3), and the data are presented as the mean ± standard deviation), (**B**) shows the heatmaps and hierarchical cluster analyses of volatile compounds in pineapple pomace wine (Euclidean distance and Ward’s method). Red: relatively high content; blue: relatively low content. (**C**) shows a diagram illustrating the aroma-producing mechanism of SC.QQT in pineapple pomace wine.

During the three distinct fermentation stages of pineapple pomace wine, a total of 61 volatile compounds were detected, comprising 9 alcohols, 18 esters, 8 acids, 4 aldehydes, 6 ketones, 3 alkanes, 5 alkenes, and 8 other volatile compounds (Table S1). A clustered heatmap was generated based on these results (Figure 4B). In contrast, the CK group samples at fermentation days 0, 7, and 14 yielded 14, 41, and 47 detected aroma compounds, respectively, while the SC group samples at fermentation days 0, 7, and 14 yielded 42, 40, and 49 detected aroma compounds, respectively. These compounds are expected to significantly influence the flavor profile of pineapple pomace wine.

Alcoholic compounds are produced by yeasts through sugar metabolism, decarboxylation reactions, and amino acid deamination [36]. These substances not only complement the ester aroma but also enhance the overall harmony of the fruit wine bouquet. Compared with the CK group, the SC group exhibited the highest alcohol content at day 0 of fermentation (52.91% ± 0.67%), followed by a gradual decline over time, likely attributable to alcohol oxidation or esterification during later fermentation stages (Figure 4A). In all tested samples, both phenethyl alcohol and 3,4-dimethylbenzyl alcohol were detected, with phenethyl alcohol present at relatively higher levels. Phenethyl alcohol, a fermentation byproduct with a rose-like fragrance, is primarily produced via the Ehrlich pathway from free phenylalanine [37]. Genome sequencing of SC.QQT predicted that increased phenethyl alcohol production is associated with the aromatic amino acid transaminases encoded by *ARO8* and *ARO9*, as well as the phenylpyruvate decarboxylase encoded by *ARO10* (Figure 4C). Based on established yeast metabolic pathways, these genes are predicted to contribute to phenethyl alcohol biosynthesis via the *Ehrlich* pathway [38]. However, direct transcriptional or enzymatic validation of their specific roles in SC.QQT was not performed in this study; functional studies are required to confirm these predictions. Additionally, the *BAT1* gene, which is associated with the *Ehrlich* pathway, is preferentially expressed during the exponential growth phase in yeast, catalyzes the transamination of branched-chain amino acids, and has been shown to increase isopentanol levels [38]. Notably, the predominant alcohol in pineapple pomace wine is (S)-(+)-1,2-propanediol. Numerous microorganisms in nature possess the ability to produce 1,2-propanediol. In addition to bacterial production, several studies in the late 1960s reported that certain yeasts (e.g., *Candida parapsilosis* and *Candida rosea*) can produce 1,2-propanediol under aerobic conditions [39]. Previously, Główka et al. [40] reported that certain *S. cerevisiae* and fungal strains can biosynthesize S-1,2-propanediol from L-rhamnose and L-fucose. This process is associated with the propanol-preferring alcohol dehydrogenase encoded by the *adhP* and *frmA* genes in the SC.QQT genome (Table S2).

Esters are formed during yeast fermentation primarily through the reaction of alcohols with acetyl-CoA, catalyzed by acetyltransferases, and can also be generated via transesterification reactions [41]. Testing revealed that ester content in both the CK group and SC group increased with extended fermentation duration. The SC group consistently exhibited a higher proportion of esters than the control group, indicating that inoculation with SC.QQT enhances volatile ester content in fruit wine, resulting in a more harmonious and balanced aroma profile (Figure 4A). Among the esters, ethyl acetate (clear and slightly fruity), ethyl 9-hexadecenoate (mild fatty and fruity), ethyl hexadecanoate (subtle waxy and buttery), ethyl dodecanoate (old-fashioned and buttery), ethyl decanoate (coconut-like aroma), ethyl caproate (rose fragrance), ethyl hexanoate (green apple scent), and ethyl nonenoate (fresh fruit and green notes) were identified. These compounds further enhance the aromatic complexity of pineapple pomace wine. In response to this characteristic, genes annotated as involved in ester synthesis, including *ATF*, *FAS1*, and *FAS2*, were identified (Table S2). The *ATF* gene is predicted to encode ethanol acetyltransferase, which may facilitate ethyl acetate formation [42]. Its expression level determines the ethyl acetate content in fermentation products. The *FAS1* and *FAS2* genes encode the β-subunit and α-subunit of fatty acid synthase, respectively [43]. These genomic associations provide a predicted biosynthetic basis for the observed ester profiles; however, functional validation is required to confirm their causal roles (Figure 4C).

Acidic compounds in fruit wine originate primarily from the fruit itself. They are not only considered aroma compounds but also serve as aroma precursors, being synthesized through various biochemical reactions [38]. Compared with the control group, the SC group exhibited overall stable acid content throughout the fermentation process, providing a relatively consistent acid source for ester synthesis (Figure 4A). Acetic acid was detected at relatively high levels across all treatment groups. During glycolysis, yeast breaks down glucose into pyruvate, which is subsequently converted into acetaldehyde under anaerobic conditions—an intermediate step in ethanol production. Due to acetaldehyde’s potent toxicity, yeast attempts to oxidize it into acetic acid to reduce its toxicity [44]. Whole-genome sequencing of SC.QQT revealed the presence of the *ACH1* gene, which is predicted to encode acetyl-CoA hydrolase (Figure 4C). This enzyme catalyzes the hydrolysis of acetyl-CoA to acetate and CoA within the mitochondria [45]. However, direct evidence linking *ACH1* expression to acetic acid levels in SC.QQT is currently lacking, and future metabolic studies are required to validate this hypothesis.

Aldehyde compounds in fruit wines are typically derived from amino acid catabolism and the auto-oxidation of unsaturated fatty acids [46]. Although aldehydes are present at relatively low concentrations, their low odor detection thresholds enable them to contribute significantly to fresh, herbal aroma notes [38]. Compared with alcohols, esters, and acids, fewer types and lower concentrations of aldehydes were detected in this study (Figure 4A), with only 14 types identified. This may be attributed to the NAD ^+^-dependent aldehyde dehydrogenase encoded by the *ALDH* gene in the SC.QQT genome (Table S2), which oxidizes aldehydes into their corresponding carboxylic acids during fermentation [47]. This enzymatic activity explains the relatively low and eventually undetectable aldehyde content in pineapple pomace wine during fermentation. Among the detected aldehydes, phthalaldehyde was present in all SC group samples, exhibiting a pronounced bitter almond odor.

Ketone compounds are formed via the degradation of lipids and amino acids during fermentation [48]. Similarly, testing revealed that ketone compounds are also relatively low in content, yet they typically possess distinctive aromas. Compounds such as 1-(2-Hydroxy-5-methylphenyl) ethanone and 1-(4-hydroxy-3,5-dimethoxyphenyl) ethanone contributed phenolic aromas to pineapple pomace wine. The SC.QQT genome harbors the associated *DLAT* gene (Figure 4C), which encodes the E2 component of pyruvate dehydrogenase. This enzyme may indirectly influence ketone production by regulating intracellular acetyl-CoA levels, though experimental validation of this pathway in SC.QQT is required [49].

Additionally, relatively low levels of other volatile compounds, such as alkanes and alkenes, were detected in pineapple pomace wine, including 1-methyl-2-(1-methylpentyl) cyclopropane, α-caryophyllene, and 2,4-di-tert-butylphenol. In summary, the flavor profile of fruit wines results from the combined effects of various volatile compounds. Inoculation with SC.QQT led to increased levels of alcohols, esters, and acids in pineapple pomace wine, shaping a distinct volatile profile. Although genomic analysis identified genes that provide a predicted biosynthetic basis for the observed volatile profiles, these associations remain hypothetical without direct experimental validation. Future studies should focus on confirming the functions of these genes to elucidate the precise mechanisms underlying aroma production in SC.QQT.

## 3. Materials and Methods

### 3.1. Growth Conditions for Fungal and Bacterial Strains

SC.QQT was isolated from pineapple pomace wine and deposited in the Guangdong Microbial Culture Collection Center (GDMCC) under accession number GDMCC 65144. SC.YT39, preserved under accession number GDMCC 2.137, was selected as the positive control. Both strains were cultured in YPD liquid medium (Qingdao High tech Industrial Park Haibo Biotechnology Co., Ltd., Qingdao, China) at 28 °C with shaking at 180 rpm for 24 h.

The bacterial strains used in the antimicrobial experiments were *E. coli* O157:H7(ATCC 35150; EC.35150, GDMCC No. 1.707), *S*. *enterica* ATCC 9120 (SE.9120, GDMCC No. 1.1114), *S*. *aureus* ATCC 25923 (SA.25923, GDMCC No. 1.174), and *L*. *monocytogenes* ATCC 19115 (LM.19115, GDMCC No. 1.347). These strains were cultured in LB broth (Guangdong Huankai Biotechnology Co., Ltd., Guangzhou, China) at 37 °C with shaking at 180 rpm for 18 h.

### 3.2. Genome Sequencing and Functional Analysis

The genome of SC.QQT was sequenced using both the DNBSEQ and PacBio platforms (BGI, Shenzhen, China). The sequencing depths were 76× for BGISEQ and 113× for PacBio. Raw reads were filtered to remove adapters and low-quality bases using SOAPnuke (v1.5.6). Genome assembly was performed with hifiasm (v0.17.4), yielding an N50 of 941,290 bp.

Quality assessment confirmed the absence of significant contamination. GC-Depth analysis revealed a normal distribution without exogenous peaks, and repeat sequence analysis indicated that repetitive elements constituted only 4.95% of the genome. Furthermore, BUSCO (v5.4.2) analysis indicated 97.95% completeness, validating the high integrity of the assembly. Non-coding RNA prediction was performed using RNAmmer (v1.2).

Comprehensive functional annotation of the predicted genes was performed using the KOG (EuKaryotic Orthologous Groups), GO (Gene Ontology), and KEGG (Kyoto Encyclopedia of Genes and Genomes) databases. KOG annotations were mapped to the KOG database (v20090331) through Diamond software (v2.1.9), GO annotations were obtained via sequence similarity-based mapping to the GO database (releases_2019–07−01) using Diamond software, and KEGG annotations were mapped to the KEGG database(v109.0) via Diamond software. Concurrently, antibiotic resistance genes in SC.QQT were systematically identified and analyzed using the CARD (v3.0.9) (Comprehensive Antibiotic Resistance Database).

### 3.3. Comparative Genome Analysis

Perform a comparative genomic analysis of the genome sequence of SC.QQT based on the reference fungal genome sequence and MUMmer (v3.22) alignment results. The reference strains include *Saccharomyces cerevisiae* Y55 (GCA_903819135.2), *Saccharomyces cerevisiae* SX2 (GCA_903819175.2), *Saccharomyces cerevisiae* BJ4 (GCA_903819145.2), *Saccharomyces cerevisiae* YJM1388 (GCA_000977505.2), and *Saccharomyces cerevisiae* IMX2600 (GCA_030292175.1).

### 3.4. Safety Analysis of the Strain

#### 3.4.1. Hemolysis Test

Following the method described in the literature [11], activated SC.QQT was streaked onto Columbia blood agar plates (Changde Beichenman Biotechnology Co., Ltd., Changde, China) and cultured at 28 °C for 48 h. Hemolytic activity was assessed by observing and photographing zones of hemolysis surrounding individual colonies. In the assay, SC.YT39 served as the negative control, and EC.35150 was used as the positive control.

#### 3.4.2. Antibiotic Resistance

The antibiotic resistance profile of SC.QQT was evaluated using the agar diffusion assay, following a previously described method with minor modifications [50]. It is important to note that while this method is widely used for bacteria, it is not a standardized clinical protocol for yeasts; herein, it was employed solely as a phenotypic screening tool to investigate the general antimicrobial tolerance of the strain. Strains SC.QQT and SC.YT39 were separately inoculated into YPD liquid medium and activated by culturing at 28 °C for 48 h on a rotary shaker (200 rpm) to achieve active growth. Subsequently, 100 µL of each well-mixed culture was evenly spread onto the surface of YPD agar plates, with SC.YT39 serving as the negative control. Commercially available antibiotic susceptibility disks (Changde Beichenman Biotechnology Co., Ltd., China) were placed aseptically onto the agar surface. After allowing 15 min for disk adsorption, the plates were incubated statically at 28 °C for 48 h. The diameter of each inhibition zone (in mm) was measured using a digital vernier caliper.

### 3.5. Probiotic Analysis of the Strain

#### 3.5.1. Acid Resistance

The acid tolerance of SC.QQT was assessed using a modified version of the method described by Zheng et al. [25]. The pH of YPD liquid medium was adjusted to 1.5, 2.0, 2.5, 3.0, 3.5, 4.0, 4.5, 5.0, 5.5, and 6.0 using 1 mol/L HCl (Sinopharm Chemical Reagent Co., Ltd., Shanghai, China), and the media were sterilized by autoclaving at 121 °C for 20 min. Strains SC.QQT and SC.YT39 were inoculated at 2% (*v*/*v*) into the prepared YPD liquid medium and pre-cultured at 28 °C for 48 h to ensure consistent activation. Subsequently, the OD_600nm_ values of each culture were measured under the respective pH conditions. SC.YT39 was included as the negative control strain. The survival rate was calculated using the following formula.

Survival rate (%) = OD_600nm_ (experiment)/OD_600nm_ (maximum) × 100

In this equation, OD_600nm_ (experiment) is the OD_600nm_ value measured after the strain has been cultured under a specific pH condition; OD_600nm_ (maximum) is the maximum OD_600nm_ value measured for that strain under optimal growth conditions.

#### 3.5.2. Bile Salt Tolerance

The bile salt tolerance of SC.QQT was determined using a modified version of the method described by Zheng et al. [25]. YPD liquid media were prepared with varying concentrations of bovine bile salts (Shanghai Boweilin Biotechnology Co., Ltd., Shanghai, China): 0.1%, 0.2%, 0.3%, 0.4%, 0.5%, 0.6%, 1.0%, 1.5%, and 2.0%. The media were sterilized by autoclaving at 121 °C for 20 min. Strains SC.QQT and SC.YT39 were inoculated separately into the prepared YPD media at a 2% inoculum size and cultured at 28 °C for 48 h. The OD_600nm_ values of the strains were measured under the different bile salt concentrations. SC.YT39 served as the negative control strain. The survival rate was calculated using the following formula.

Survival rate (%) = OD_600nm_ (experiment) / OD_600nm_ (maximum) × 100

In this equation, OD_600nm_ (experiment) is the OD_600nm_ value measured after the strain has been cultured under a specific bile salt concentration; OD_600nm_ (maximum) is the maximum OD_600nm_ value measured for that strain under optimal growth conditions.

#### 3.5.3. Artificial Gastrointestinal Fluid Tolerance

The method for preparing artificial gastric fluid was as follows: Add 0.3% (*w*/*v*) pepsin (Shanghai Macklin Biochemical Technology Co., Ltd., Shanghai, China) to the PBS buffer, adjust the solution to pH 2.5 with 1 mol/L HCl, and then filter and sterilize the solution.

The method for preparing artificial intestinal fluid was as follows: Add 0.1% (*w*/*v*) trypsin (Shanghai Macklin Biochemical Technology Co., Ltd., China) and 0.3% (*w*/*v*) bile salt to the PBS buffer, adjust the solution to pH 8.0 with sodium hydroxide (Sinopharm Chemical Reagent Co., Ltd., China), and then filter and sterilize the solution.

The simulated artificial gastrointestinal fluid experiment was conducted following the methodology of Yang et al. [51], with slight modifications. Strains SC.QQT and SC.YT39 were inoculated at 2% into YPD liquid medium and activated by incubation at 28 °C for 48 h. The cultures were centrifuged at 3000 rpm for 10 min using a refrigerated centrifuge. After discarding the supernatant, the pellets were washed twice with sterile PBS, and the bacterial suspension was adjusted to a concentration of 10^8^ CFU/mL. One milliliter of the bacterial suspension was washed twice and then mixed with 9 mL of simulated gastric fluid, followed by static incubation at 28 °C for 3 h. Subsequently, 1 mL of the simulated gastric fluid mixture was combined with 9 mL of simulated intestinal fluid and incubated statically at 28 °C for 3 h. Viable bacterial counts were determined at the beginning (0 h) and end (3 h) of the incubation periods in both the simulated gastric and intestinal fluids to evaluate the tolerance of SC.QQT to the simulated gastrointestinal conditions. SC.YT39 served as the negative control strain. The survival rate was calculated using the following formula.

Survival rate (%) = (N_m_/N_n_) × 100

In this equation, N_m_ represents the viable bacterial count after 3 h in simulated gastrointestinal fluid, and N_n_ represents the viable bacterial count at 0 h in simulated gastrointestinal fluid.

#### 3.5.4. Antibacterial Test

The antibacterial activity of the SC.QQT stock solution and its corresponding bacterial suspension against four indicator strains was evaluated using the agar well diffusion method with slight modifications [52]. The cell densities of the indicator strains (EC.35150, SE.9120, SA.25923, and LM.19115) were adjusted to an OD_600nm_ of 0.5. Each suspension was then mixed with molten LB agar (cooled to ~45 °C) and poured into sterile Petri dishes to form seeded agar plates. After solidification, wells (7 mm in diameter) were aseptically punched into the agar using a sterile pipette tip. Subsequently, 90 µL of either the SC.QQT stock solution or its bacterial suspension was loaded into each well. Plates were incubated at 37 °C for 24 h, and the diameters of the resulting inhibition zones were measured using a digital vernier caliper. Sterile distilled water was included as a blank control, while strain SC.YT39 served as the negative control.

### 3.6. Analysis of Aroma-Producing Characteristics of Strains

Volatile components in pineapple pomace wine samples were analyzed using headspace solid-phase microextraction coupled with gas chromatography–mass spectrometry (SPME-GC-MS) (Agilent 8890B, Agilent Technologies Inc., Santa Clara, CA, USA) at three time points: before fermentation (0 d), mid-fermentation stage (7 d), and after fermentation completion (14 d). For each analysis, 5 mL of sample was precisely transferred into a 20 mL headspace vial and extracted at 80 °C for 40 min. A 50/30 μm DVB/CAR/PDMS fiber (Supelco, Bellefonte, PA, USA) was inserted into the injection port (maintained at 250 °C) and desorbed for 5 min. The chromatographic conditions were as follows: the oven temperature was programmed to increase at a rate of 5 °C/min to 220 °C, where it was held for 15 min; the initial column temperature was set at 50 °C; the injection was performed in splitless mode. Electron ionization (EI) was employed with an ion source temperature of 220 °C and an interface temperature of 240 °C. Helium was used as the carrier gas. Compound identification was achieved by comparing mass spectra with the NIST 20 and Wiley 11 databases, accepting matches with a similarity index greater than 80%. The relative content of each volatile compound was determined using the peak area normalization method, providing a semi-quantitative analysis of the overall volatile profile. Naturally fermented samples (CK) served as the control group, while samples inoculated with SC.QQT (SC) constituted the experimental group.

### 3.7. Data Analysis

All experiments were performed in triplicate (*n* = 3), and the data are presented as the mean ± standard deviation (SD). Statistical analysis was conducted using IBM SPSS Statistics 26 software. One-way analysis of variance (ANOVA) was applied to determine significant differences among groups, followed by multiple comparisons using Duncan’s method. Differences were considered statistically significant at a significance level of *p <* 0.05. Graphs were generated using Origin 2024 and Wekemo Bioincloud software (https://www.bioincloud.tech, accessed on 24 May 2026).

## 4. Conclusions

This study integrated genomic and phenotypic analyses to comprehensively evaluate the safety, probiotic potential, and aroma-producing characteristics of SC.QQT, a strain isolated from pineapple pomace wine. The strain exhibited no hemolytic activity, carried only intrinsic antibiotic resistance genes with a low risk of horizontal transfer, and met food safety requirements. It demonstrated robust tolerance to acidic conditions, bile salts, and simulated gastrointestinal fluid, indicating its potential suitability for probiotic applications, subject to in vivo validation. Furthermore, SC.QQT was associated with an altered volatile profile, including increased levels of alcohols and esters, which correlated with the presence of predicted aroma-related genes in the genome assembly. Collectively, SC.QQT passed basic safety screenings and represents a functionally stable strain with promising probiotic-related traits and notable metabolic capabilities inferred from the genome assembly. Although its genomic and phenotypic characteristics suggest potential for pilot-scale fermentation trials in fruit wine processing, further industrial validation is required to confirm its commercial feasibility. Future studies should focus on in vivo functional verification and optimization of industrial fermentation processes. All genomic interpretations presented in this study are derived from assembly based predictions and lack experimental validation; therefore, they should be regarded as preliminary and hypothesis-generating.

## Figures and Tables

**Table 1:** Genomic characteristics of SC.QQT.

Attributes	Value
Genome size	30,256,254 bp
Total gene length	22,062,659 bp
Total number of genes	12,899
GC content	37.30%
Number of tRNA genes	703
Number of rRNA genes	159
Number of sRNA genes	117
Number of snRNA genes	222
Number of miRNA genes	411

**Table 2:** Antibiotic sensitivity of SC.QQT.

Antibiotic Name	Dosage μg/Tablet	SC.QQT Colony Diameter	Sensitivity	SC.YT39 Colony Diameter	Sensitivity
Penicillin	10	ND	R	ND	R
Chloramphenicol	30	ND	R	ND	R
Streptomycin	10	ND	R	ND	R
Erythromycin	15	ND	R	ND	R
Tetracycline	30	ND	R	ND	R
Ciprofloxacin	30	ND	R	ND	R
Levofloxacin	5	ND	R	ND	R
Polymyxin B	300	ND	R	ND	R
Norfloxacin	10	ND	R	ND	R
Cefuroxime sodium	30	ND	R	ND	R
Ceftriaxone	30	ND	R	ND	R
Phenoxymethylpenicillin	10	ND	R	ND	R
Amikacin	30	ND	R	ND	R
Kanamycin	30	ND	R	ND	R
Minocycline	30	ND	R	ND	R
Gentamicin	10	ND	R	ND	R
Ampicillin	10	ND	R	ND	R
Pivalicillin	100	ND	R	ND	R
Enrofloxacin	5	ND	R	ND	R
Azithromycin	15	ND	R	ND	R
Lincomycin	2	ND	R	ND	R
Cefalexin	30	ND	R	ND	R
Co-trimoxazole	25	ND	R	ND	R
Cefoperazone	75	ND	R	ND	R
Doxycycline	30	ND	R	ND	R
Cefotaxime	30	ND	R	ND	R
Florfenicol	30	ND	R	ND	R
Clindamycin	2	ND	R	ND	R
Cefazolin	30	ND	R	ND	R
Vancomycin	30	ND	R	ND	R
Imipenem	10	ND	R	ND	R

Note: “ND” denotes no inhibition zone and “R” indicates resistant.

## Data Availability

Data available in a publicly accessible repository. The genome sequence data have been deposited in the NCBI database under BioProject (PRJNA1452283) and BioSample (SAMN57228753). These data are currently undergoing processing and will be publicly released upon acceptance of the manuscript. The original contributions presented in this study are included in the article. Further inquiries can be directed to the corresponding authors.

## Supplementary Materials

The following supporting information can be downloaded at: https://www.mdpi.com/article/10.3390/molecules31132310/s1, Table S1. Analysis of volatile compounds in pineapple pomace wine; Table S2. Functional genes of the SC.QQT genome; Table S3. Genome annotation of SC.QQT.
