# Supplementary material for: Genomic and Phenotypic Evaluation of Safety, Probiotic Potential, and Aroma Production of Saccharomyces cerevisiae FOSU-QQT
Source: Molecules. 2026 Jul 1;31(13):2310. doi: 10.3390/molecules31132310 (PMC13363411; doi:10.3390/molecules31132310)
Supplement: Supplementary file 1 [file molecules-31-02310-s001.zip › Supplementary material (1).pdf]

Table S1. Analysis of volatile compounds in pineapple pomace wine

No.	RT (min)	Substance name	Relative content (%)					
			CK			SC.QQT		
			0d	7d	14d	0d	7d	14d
		**Alcohols**						
1	12.987	1-Butanol, 3-methyl-	0.85 ± 0.05^{Ab}	ND	3.33 ± 0.32^{Ba}	ND	1.10 ± 0.12^{Ab}	3.99 ± 0.19^{Aa}
2	30.387	Phenylethyl Alcohol	1.68 ± 0.11^{Bb}	8.37 ± 0.05^{Ba}	11.47 ± 0.47^{Aa}	13.57 ± 3.13^{Aa}	17.15 ± 0.74^{Aa}	11.17 ± 3.85^{Aa}
3	34.365	3,4-Dimethylbenzyl alcohol	0.19 ± 0.02^{Ab}	0.20 ± 0.11^{Ab}	8.99 ± 0.49^{Aa}	0.13 ± 0.02^{Ba}	0.09 ± 0.07^{Aa}	0.08 ± 0.06^{Ba}
4	22.451	2,3-Butanediol	ND	0.18 ± 0.14^{Ab}	8.58 ± 0.84^{Aa}	ND	ND	1.47 ± 0.13^{Ba}
5	10.319	1-Propanol, 2-methyl-	ND	0.23 ± 0.22^{Aa}	0.35 ± 0.12^{Aa}	0.05 ± 0.05^{Ab}	0.33 ± 0.18^{Aa}	0.19 ± 0.06^{Aab}
6	30.241	1-Decanol	ND	13.83 ± 0.55^{Aa}	0.02 ± 0.01^{Ab}	ND	ND	0.01 ± 0.00^{Aa}
7	42.328	1H-Inden-5-ol, 2,3-dihydro-	ND	ND	0.05 ± 0.03^{Aa}	0.05 ± 0.02^{Ab}	0.10 ± 0.01^{Aa}	0.02 ± 0.01^{Ac}
8	24.395	1-Nonanol	ND	ND	8.59 ± 0.65^{Aa}	0.02 ± 0.01^{Aa}	ND	0.01 ± 0.01^{Ba}
9	7.433	(S)-(+)-1,2-Propanediol	ND	ND	ND	39.09 ± 0.87^{Aa}	ND	14.77 ± 0.66^{Ab}
		Total	2.72 ± 0.17^{Bc}	22.81 ± 0.33^{Ab}	41.38 ± 0.25^{Aa}	52.91 ± 0.67^{Aa}	18.77 ± 0.94^{Ac}	31.71 ± 0.34^{Ab}
		**Esters**						
10	6.804	Ethyl Acetate	0.25 ± 0.13^{Bb}	4.38 ± 0.97^{Aa}	6.74 ± 0.50^{Aa}	1.39 ± 0.40^{Ab}	4.00 ± 0.05^{Aa}	4.45 ± 0.19^{Ba}
11	43.488	Ethyl 18-nonadecenoate	ND	0.05 ± 0.02^{Ab}	9.03 ± 0.56^{Aa}	ND	ND	ND
12	41.033	Ethyl Oleate	ND	0.10 ± 0.01^{Ab}	0.58 ± 0.16^{Aa}	0.03 ± 0.01^{Ab}	0.10 ± 0.03^{Ab}	0.41 ± 0.06^{Aa}
13	40.379	Octadecanoic acid, ethyl ester	ND	0.09 ± 0.06^{Aa}	0.30 ± 0.12^{Aa}	0.04 ± 0.02^{Ab}	0.14 ± 0.02^{Aa}	0.23 ± 0.08^{Aa}
14	37.562	Ethyl 2-hydroxy-3-phenylpropanoate	ND	0.17 ± 0.10^{Aa}	0.32 ± 0.23^{Aa}	ND	0.11 ± 0.03^{Aa}	0.15 ± 0.11^{Aa}
15	37.179	Ethyl 9-hexadecenoate	ND	0.25 ± 0.14^{Aa}	0.39 ± 0.17^{Aa}	0.06 ± 0.01^{Ab}	0.18 ± 0.03^{Ab}	0.70 ± 0.37^{Aa}
16	36.641	Hexadecanoic acid, ethyl ester	0.03 ± 0.01^{Bb}	1.01 ± 0.40^{Ab}	3.88 ± 0.98^{Aa}	0.19 ± 0.05^{Ab}	0.65 ± 0.02^{Bb}	3.05 ± 0.85^{Aa}
17	32.798	Tetradecanoic acid, ethyl ester	ND	0.19 ± 0.10^{Aa}	0.15 ± 0.14^{Ba}	0.08 ± 0.03^{Ab}	0.09 ± 0.04^{Ab}	0.50 ± 0.33^{Aa}

18	28.599	Dodecanoic acid, ethyl ester	ND	0.35 ± 0.09^{Ab}	0.89 ± 0.07^{Ba}	0.05 ± 0.03^{Ab}	0.33 ± 0.02^{Ab}	3.19 ± 0.61^{Aa}
19	23.994	Decanoic acid, ethyl ester	ND	0.42 ± 0.07^{Ab}	3.36 ± 0.93^{Aa}	0.23 ± 0.06^{Ab}	0.38 ± 0.04^{Ab}	2.53 ± 0.84^{Ba}
20	18.984	Octanoic acid, ethyl ester	ND	0.97 ± 0.53^{Aa}	1.54 ± 0.40^{Aa}	1.93 ± 0.59^{Aa}	1.08 ± 0.31^{Aa}	1.49 ± 0.62^{Aa}
21	13.750	Hexanoic acid, ethyl ester	ND	0.28 ± 0.25^{Aa}	0.27 ± 0.16^{Aa}	0.60 ± 0.17^{Aa}	0.66 ± 0.03^{Aa}	0.54 ± 0.24^{Aa}
22	10.988	1-Butanol, 3-methyl-, acetate	ND	0.47 ± 0.27^{Ba}	ND	ND	1.16 ± 0.06^{Aa}	1.19 ± 0.54^{Aa}
23	25.129	Ethyl nonenoate	ND	0.05 ± 0.04^{Aa}	0.03 ± 0.01^{Aa}	0.23 ± 0.11^{Aa}	0.02 ± 0.01^{Ab}	0.07 ± 0.06^{Ab}
24	21.545	Nonanoic acid, ethyl ester	ND	0.15 ± 0.09^{Aa}	0.07 ± 0.03^{Aa}	0.27 ± 0.03^{Aa}	0.12 ± 0.04^{Ab}	0.08 ± 0.07^{Ab}
25	31.792	Acetic acid, 2-phenylethyl ester	ND	ND	1.70 ± 0.99^{Ba}	0.75 ± 0.18^{Ab}	3.64 ± 0.98^{Aa}	3.39 ± 0.76^{Aa}
26	38.458	Heptadecanoic acid, ethyl ester	ND	ND	1.57 ± 0.27^{Aa}	ND	ND	0.03 ± 0.01^{Ba}
27	9.180	Butanoic acid, ethyl ester	ND	ND	ND	0.03 ± 0.02^{Aa}	0.02 ± 0.01^{Aa}	0.02 ± 0.02^{Aa}
		Total	0.28 ± 0.14^{Bc}	8.92 ± 0.55^{Ab}	27.55 ± 0.97^{Aa}	5.10 ± 0.44^{Ac}	9.02 ± 0.79^{Ab}	22.02 ± 0.82^{Ba}
		**Acids**						
28	19.617	Acetic acid	2.04 ± 0.20^{Ab}	5.59 ± 0.99^{Aa}	6.52 ± 0.87^{Aa}	0.32 ± 0.05^{Ba}	0.21 ± 0.02^{Ba}	0.22 ± 0.08^{Ba}
29	37.124	n-Decanoic acid	0.09 ± 0.06^{Ab}	0.72 ± 0.32^{Aab}	0.95 ± 0.48^{Aa}	0.05 ± 0.01^{Ab}	0.09 ± 0.06^{Bab}	0.20 ± 0.09^{Aa}
30	35.202	Nonanoic acid	0.04 ± 0.01^{Aa}	0.10 ± 0.07^{Aa}	ND	0.06 ± 0.02^{Aa}	0.03 ± 0.02^{Aa}	0.04 ± 0.03^{Aa}
31	13.061	Propanoic acid, anhydride	0.23 ± 0.10^{Bb}	0.97 ± 0.47^{Bab}	1.67 ± 0.89^{Aa}	1.96 ± 0.49^{Aa}	1.98 ± 0.11^{Aa}	2.13 ± 0.37^{Aa}
32	41.085	Dodecanoic acid	ND	0.10 ± 0.07^{Aa}	0.05 ± 0.04^{Aa}	ND	0.03 ± 0.01^{Bb}	0.10 ± 0.02^{Aa}
33	33.164	Octanoic acid	ND	1.71 ± 0.95^{Aa}	0.19 ± 0.18^{Ab}	0.66 ± 0.18^{Aa}	0.26 ± 0.03^{Bb}	0.49 ± 0.14^{Aab}
34	28.831	Pentanoic acid	ND	0.20 ± 0.10^{Aa}	0.09 ± 0.01^{Ba}	0.33 ± 0.08^{Aa}	0.37 ± 0.18^{Aa}	0.27 ± 0.05^{Aa}
35	28.835	Hexanoic acid	ND	ND	ND	0.31 ± 0.08^{Aa}	0.31 ± 0.03^{Aa}	ND
		Total	2.40 ± 0.33^{Bb}	9.39 ± 0.95^{Aa}	9.47 ± 0.76^{Aa}	3.69 ± 0.30^{Aa}	3.28 ± 0.25^{Ba}	3.45 ± 0.50^{Ba}
		**Aldehydes**						

36	21.798	Benzaldehyde	0.10±0.03[Aa]	0.10±0.03[Aa]	0.06±0.05[Aa]	ND	ND	ND
37	28.612	Benzaldehyde, 3,4-dimethyl-	0.33±0.02[Aa]	ND	ND	0.28±0.08[Aa]	ND	ND
38	28.609	Isophthalaldehyde	ND	0.43±0.09[Aa]	ND	0.27±0.04[Aa]	0.18±0.09[Bab]	0.07±0.04[Ab]
39	32.054	Benzaldehyde, 2,5-dimethyl-	ND	ND	0.13±0.08[Aa]	ND	ND	0.23±0.14[Aa]
		Total	0.43±0.04[Ba]	0.53±0.06[Aa]	0.19±0.08[Ab]	0.55±0.04[Aa]	0.18±0.09[Bb]	0.30±0.14[Ab]
		**Ketones**						
40	32.283	2H-Pyran-2,6(3H)-dione	0.14±0.23[Aa]	ND	ND	0.09±0.02[Aa]	0.09±0.01[Aa]	ND
41	23.490	Fluridone	0.03±0.01[Aa]	0.03±0.00[Aa]	0.05±0.04[Aa]	ND	ND	0.03±0.01[Aa]
42	37.182	1-Heptanone, 1-(2-furanyl)-	ND	0.28±0.07[Aa]	ND	0.02±0.01[Aa]	ND	ND
43	36.006	Ethanone, 1-(2-hydroxy-5-methylphenyl)-	ND	1.37±0.45[Aa]	3.35±0.95[Aa]	ND	ND	ND
44	36.557	Ethanone, 1-(4-hydroxy-3,5-dimethoxyphenyl)-	ND	0.13±0.11[Aa]	ND	0.02±0.02[Aa]	0.20±0.10[Aa]	0.20±0.17[Aa]
45	13.047	Methyl vinyl ketone	ND	ND	0.48±0.10[Aa]	0.25±0.09[Aa]	ND	ND
		Total	0.17±0.03[Bb]	1.81±0.60[Ab]	3.88±0.94[Aa]	0.38±0.10[Aa]	0.29±0.11[Ba]	0.23±0.16[Ba]
		**Alkanes**						
46	24.398	Cyclopropane, 1-methyl-2-(1-methylpentyl)-	ND	ND	ND	0.04±0.03[Aa]	ND	0.01±0.00[Aa]
47	26.369	Hexadecane	ND	0.10±0.05[Aa]	ND	0.05±0.04[Aa]	ND	0.03±0.02[Aa]
48	40.760	Heptane, 3,3,4-trimethyl-	ND	ND	0.15±0.09[Aa]	ND	ND	0.01±0.00[Ba]
		Total	0.00	0.10±0.05[Aa]	0.15±0.09[Aa]	0.09±0.08[Aa]	0.00	0.05±0.02[Aa]
		**Olefins**						
49	18.311	(3Z,5E)-1,3,5-Undecatriene	ND	0.04±0.01[Aa]	0.05±0.02[Aa]	0.15±0.09[Aa]	0.27±0.10[Ba]	0.05±0.03[Aa]

50	21.936	(-)-Aristolene	ND	ND	0.02±0.01^{Aa}	ND	ND	0.01±0.01^{Aa}
51	23.248	1,3,5,8-Undecatetraene	ND	ND	0.05±0.01^{Aa}	ND	ND	0.06±0.02^{Aa}
52	28.908	.gamma.-Muurolene	ND	ND	0.04±0.03^{Aa}	0.10±	ND	0.02±0.00^{Aa}
53	34.088	.alpha.-Calacorene	ND	ND	2.56±0.87^{Aa}	0.05±0.04^{Aa}	0.01±0.01^{Aa}	ND
		Total	0.00	0.04±0.01^{Bb}	2.72±0.88^{Aa}	0.30±0.13^{Aa}	0.28±0.38^{Aa}	0.14±0.03^{Ba}
		**Others**						
54	37.656	2,4-Di-tert-butylphenol	6.88±0.50^{Aa}	11.26±0.88^{Aa}	9.83±0.78^{Aa}	2.46±0.25^{Bb}	8.87±0.70^{Aa}	7.74±0.56^{Aa}
55	36.717	Naphthalene, 1,6-dimethyl-4-(1-methylethyl)-	ND	0.08±0.01^{Aa}	ND	ND	0.02±0.02^{Ba}	ND
56	32.812	Phenol, 4-ethyl-2-methoxy-	ND	3.21±0.39^{Aa}	2.08±0.72^{Aa}	0.53±0.11^{Ba}	ND	ND
57	7.606	1,3-Dioxolane, 2,4,5-trimethyl-	ND	0.16±0.06^{Aa}	0.33±0.11^{Aa}	ND	0.14±0.01^{Aa}	0.12±0.01^{Ba}
58	39.464	Benzofuran, 2,3-dihydro-	ND	0.20±0.09^{Ba}	0.17±0.13^{Ba}	0.48±0.10^{Ac}	0.79±0.07^{Aa}	0.63±0.02^{Ab}
59	35.447	2,3,5,6-Tetramethyl-para-phenylenediamine	ND	0.10±0.05^{Aa}	0.13±0.03^{Aa}	ND	ND	ND
60	41.806	Glycerin	ND	ND	0.48±0.12^{Aa}	ND	0.08±0.04^{Ab}	0.43±0.11^{Aa}
61	22.725	3H-pyrrole, 2-(4-methoxyphenyl)-3,4,5-triphenyl-	ND	ND	ND	0.02±0.00^{Aa}	0.04±0.05^{Aa}	0.01±0.01^{Aa}
		Total	6.88±0.50^{Ab}	15.01±0.85^{Aa}	13.02±0.90^{Aab}	3.49±0.43^{Bb}	9.94±0.59^{Ba}	8.93±0.55^{Ba}

Note: "ND" indicates that the compound was not detected. Different uppercase letters indicate significant differences between groups, while different lowercase letters indicate significant differences between the experimental groups and the control group ($p < 0.05$).

Table S2. Functional genes of the SC.QQT genome

Gene ID	Gene name	Function
**pH stress resistance**		
GME10205_g	*ATPEF0D, ATP5H, ATP7*	F-type H^+-transporting ATPase subunit d
GME10577_g	*ATPEF1D, ATP5D, ATP16*	F-type H^+-transporting ATPase subunit delta
GME11024_g	*ATPEF0B, ATP5F1, ATP4*	F-type H^+-transporting ATPase subunit b
GME11597_g	*TIM11, ATP21*	F-type H^+-transporting ATP synthase subunit e
GME1812_g	*ATPEF1B, ATP5B, ATP2*	F-type H^+-transporting ATPase subunit beta
GME3514_g	*ATPEF1A, ATP5A1, ATP1*	F-type H^+-transporting ATPase subunit alpha
GME3960_g	*ATPEF1G, ATP5C1, ATP3*	F-type H^+-transporting ATPase subunit gamma
GME4244_g	*ATPEFG, ATP5L, ATP20*	F-type H^+-transporting ATPase subunit g
GME5182_g	*ATPEFF, ATP17*	F-type H^+-transporting ATPase subunit f
GME549_g	*ATPEFH, ATP14*	F-type H^+-transporting ATPase subunit h
GME6228_g	*ATPEF0O, ATP5O, ATP5*	F-type H^+-transporting ATPase subunit O
GME6857_g	*ATPEFK, ATP19*	F-type H^+-transporting ATPase subunit k
GME7982_g	*ATPEF0C, ATP5G, ATP9*	F-type H^+-transporting ATPase subunit c
GME10263_g	*ATPEV1C, ATP6C*	V-type H^+-transporting ATPase subunit C
GME10413_g	*ATPEV0D, ATP6D*	V-type H^+-transporting ATPase subunit d
GME11125_g	*ATPEV1B, ATP6B*	V-type H^+-transporting ATPase subunit B
GME11911_g	*ATPEV0A, ATP6N*	V-type H^+-transporting ATPase subunit a
GME12625_g	*ATPEV0C, ATP6L*	V-type H^+-transporting ATPase 16kDa proteolipid subunit
GME12646_g	*ATPEV1D, ATP6M*	V-type H^+-transporting ATPase subunit D
GME12831_g	*ATPEV1G, ATP6G*	V-type H^+-transporting ATPase subunit G
GME12845_g	*ATPEV0B, ATP6F*	V-type H^+-transporting ATPase 21kDa proteolipid subunit
GME4228_g	*ATPEV1H*	V-type H^+-transporting ATPase subunit H
GME4761_g	*ATPEV1F, ATP6S14*	V-type H^+-transporting ATPase subunit F
GME5117_g	*ATPEV1E, ATP6E*	V-type H^+-transporting ATPase subunit E
GME6639_g	*ATPEV1A, ATP6A*	V-type H^+-transporting ATPase subunit A
GME190_g	*NHA1, SOD2*	sodium/hydrogen antiporter
GME9609_g	*NHA1, SOD2*	sodium/hydrogen antiporter
GME3247_g	*CHX, KHA, nhaS4*	K^+:H^+ antiporter
GME9007_g	*CHX, KHA, nhaS4*	K^+:H^+ antiporter
GME10015_g	*chaA, CAX*	Ca^{2+}:H^+ antiporter
GME11157_g	*chaA, CAX*	Ca^{2+}:H^+ antiporter

GME12240_g	*TMEM165, GDT1*	Ca^{2+}/H^+ antiporter, TMEM165/GDT1 family
GME12262_g	*chaA, CAX*	$Ca^{2+}:H^+$ antiporter
GME6590_g	*chaA, CAX*	$Ca^{2+}:H^+$ antiporter
GME886_g	--	Alkaline-phosphatase-like, core domain superfamily
GME1052_g	--	Alkaline-phosphatase-like, core domain superfamily
GME1074_g	--	Alkaline-phosphatase-like, core domain superfamily
GME1099_g	--	Alkaline-phosphatase-like, core domain superfamily
GME1272_g	--	Alkaline-phosphatase-like, core domain superfamily
GME1080_g	--	Alkaline-phosphatase-like, core domain superfamily
GME1516_g	--	Alkaline-phosphatase-like, core domain superfamily
GME4340_g	--	Alkaline ceramidase
GME3833_g	--	Alkaline ceramidase
GME12245_g	--	Alkaline ceramidase
GME11029_g	--	Alkaline ceramidase
**Bile salt stress resistance**		
GME7954_g	--	ABC transporter, ATP-binding protein
GME1753_g	--	ABC transporter, ATP-binding protein
GME1262_g	--	ABC transporter substrate binding protein
GME1362_g	--	ABC transporter substrate-binding protein family 5
GME1624_g	--	ABC transporter, substratebinding protein
GME1659_g	--	ABC transporter, substratebinding protein
GME1660_g	--	ABC transporter, substratebinding protein
GME10963_g	--	response to salt stress
GME11170_g	--	cellular response to salt stress
GME11356_g	--	response to salt stress
GME2353_g	--	cellular response to salt stress
GME6722_g	--	cellular response to salt stress
GME10963_g	--	response to salt stress
GME11170_g	--	cellular response to salt stress
**Osmotic stress**		
GME7502_g	--	May participate in the regulation of osmotic pressure changes within the cell
GME917_g	--	May participate in the regulation of osmotic pressure changes within the cell
GME1557_g	*opuBCD*	ABC-type proline glycine betaine transport system permease component
GME7610_g	*opuBCD*	ABC-type proline glycine betaine transport system permease component
**Adhesion**		
GME5921_g	--	cell adhesion involved in multi-species biofilm formation
GME5921_g	--	cell adhesion involved in multi-species biofilm formation
GME1512_g	*SCPA*	Segregation and condensation protein A
GME7252_g	*SCPB*	Chromosome segregation/condensation protein ScpB

GME6698_g	--	biological adhesion
GME8177_g	--	biological adhesion
GME8905_g	*Tuf,TUFM*	elongation factor Tu
GME11957_g	*GAPDH, gapA*	glyceraldehyde 3-phosphate dehydrogenase (phosphorylating)
GME3161_g	*GAPDH, gapA*	glyceraldehyde 3-phosphate dehydrogenase (phosphorylating)
GME6443_g	*TPI, tpiA*	triosephosphate isomerase (TIM)
GME7675_g	*TPI, tpiA*	triosephosphate isomerase (TIM)
GME9781_g	*TPI, tpiA*	triosephosphate isomerase (TIM)
GME12816_g	*PPIB, ppiB*	peptidyl-prolyl cis-trans isomerase B (cyclophilin B)
GME6222_g	*PPIB, ppiB*	peptidyl-prolyl cis-trans isomerase B (cyclophilin B)
GME9753_g	*PPIB, ppiB*	peptidyl-prolyl cis-trans isomerase B (cyclophilin B)
**Heat stress resistance**		
GME11776_g	*HSP110*	heat shock protein 110kDa
GME3845_g	*HSP110*	heat shock protein 110kDa
GME4358_g	*HSP110*	heat shock protein 110kDa
GME8595_g	*HSP110*	heat shock protein 110kDa
GME4061_g	*HSP70*	Belongs to the heat shock protein 70 family
GME49_g	*HSP70*	Belongs to the heat shock protein 70 family
GME5880_g	*HSP70*	Belongs to the heat shock protein 70 family
GME3931_g	--	cellular response to heat
GME5204_g	--	Heat shock protein 9/12
GME7357_g	*HSP20*	Belongs to the small heat shock protein (HSP20) family
GME6299_g	--	Activator of 90 kDa heat shock protein ATPase homolog
GME8216_g	*HSP26, HSP42*	Molecular chaperone (small heat-shock protein Hsp26/Hsp42)
GME12351_g	--	Heat shock transcription factor
GME3111_g	--	HEAT repeat-containing protein
GME10819_g	*dnaK, HSPA9*	molecular chaperone DnaK
GME11801_g	*GRPE*	molecular chaperone GrpE
GME1376_g	*dnaJ*	molecular chaperone DnaJ
GME928_g	--	response to heat
**Cold stress resistance**		
GME1034_g	--	Cold shock domain
GME1610_g	--	Cold shock domain
GME7843_g	--	Cold-shock (CSD) domain
GME1644_g	--	Cold-shock protein, DNA-binding
GME1989_g	--	Cold-shock protein, DNA-binding
GME7460_g	--	Cold-shock protein, DNA-binding

GME1781_g	--	RNase II/RNase R, cold shock domain
GME9486_g	*DIS3*	Dis3-like cold-shock domain 2
GME6233_g	*DIS3*	Dis3-like cold-shock domain 2
GME6818_g	*RRP44*	Rrp44-like cold shock domain
**Oxidative stress**		
GME1045_g	*GSR, gor*	glutathione reductase (NADPH)
GME4344_g	*GSR, gor*	glutathione reductase (NADPH)
GME9366_g	*GSR, gor*	glutathione reductase (NADPH)
GME10745_g	*trxB, TRR*	thioredoxin reductase (NADPH)
GME1089_g	*trxB, TRR*	thioredoxin reductase (NADPH)
GME12769_g	*trxB, TRR*	thioredoxin reductase (NADPH)
GME297_g	*trxB, TRR*	thioredoxin reductase (NADPH)
GME6177_g	*trxB, TRR*	thioredoxin reductase (NADPH)
GME7514_g	*trxB, TRR*	thioredoxin reductase (NADPH)
GME10215_g	*GPX*	peroxiredoxin
GME11587_g	*GPX*	peroxiredoxin
GME11588_g	*GPX*	peroxiredoxin
GME11679_g	*GPX*	peroxiredoxin
GME3780_g	*GPX*	peroxiredoxin
GME7754_g	*GPX*	peroxiredoxin
GME7990_g	*GPX*	peroxiredoxin
GME10215_g	*GPX*	peroxiredoxin
GME11587_g	*GPX*	glutathione peroxidase
GME11588_g	*GPX*	glutathione peroxidase
GME11679_g	*GPX*	glutathione peroxidase
GME767_g	*GPX*	glutathione peroxidase
GME4818_g	*katE, CAT, catB, srpA*	catalase
GME5694_g	*katE, CAT, catB, srpA*	catalase
GME6262_g	*katE, CAT, catB, srpA*	catalase
GME1030_g	--	Organic hydroperoxide resistance protein famiy
GME1611_g	--	Alkyl hydroperoxide reductase subunit C/ Thiol specific antioxidant
GME11391_g	--	Cytochrome c oxidase subunit IV family
GME11751_g	*CtaG,Cox11*	Cytochrome c oxidase assembly protein CtaG/Cox11
GME10131_g	--	Haem peroxidase
**Proteases and chaperones**		
GME1095_g	*clpP, CLPP*	ATP-dependent Clp protease, protease subunit
GME1193_g	*clpB*	ATP-dependent Clp protease ATP-binding subunit ClpB
GME12319_g	*clpB*	ATP-dependent Clp protease ATP-binding subunit ClpB
GME1517_g	*clpB*	ATP-dependent Clp protease ATP-binding subunit ClpB
GME47_g	*clpB*	ATP-dependent Clp protease ATP-binding subunit ClpB
GME5633_g	*clpB*	ATP-dependent Clp protease ATP-binding subunit ClpB

GME6260_g	*clpB*	ATP-dependent Clp protease ATP-binding subunit ClpB
GME7518_g	*clpB*	ATP-dependent Clp protease ATP-binding subunit ClpB
GME7696_g	*clpB*	ATP-dependent Clp protease ATP-binding subunit ClpB
GME810_g	*clpB*	ATP-dependent Clp protease ATP-binding subunit ClpB
GME11662_g	*clpX, CLPX*	ATP-dependent Clp protease ATP-binding subunit ClpX
GME7964_g	*clpX, CLPX*	ATP-dependent Clp protease ATP-binding subunit ClpX
GME1070_g	*groES, HSPE1*	chaperonin GroES
GME6856_g	*groES, HSPE1*	chaperonin GroES
GME9905_g	*groES, HSPE1*	chaperonin GroES
GME1071_g	*groEL, HSPD1*	chaperonin GroEL
GME11115_g	*groEL, HSPD1*	chaperonin GroEL
GME3956_g	*groEL, HSPD1*	chaperonin GroEL
GME577_g	*groEL, HSPD1*	chaperonin GroEL
GME7896_g	*groEL, HSPD1*	chaperonin GroEL
GME8323_g	*groEL, HSPD1*	chaperonin GroEL
GME10819_g	*dnaK, HSPA9*	molecular chaperone DnaK
GME11801_g	*GRPE*	molecular chaperone GrpE
GME1376_g	*dnaJ*	molecular chaperone DnaJ
GME12190_g	*REC8*	meiotic recombination protein REC8, fungi type
GME4255_g	*REC8*	meiotic recombination protein REC8, fungi type
GME2810_g	*BLM, RECQL3, SGS1*	bloom syndrome protein
GME8766_g	*BLM, RECQL3, SGS1*	bloom syndrome protein
GME9680_g	*BLM, RECQL3, SGS1*	bloom syndrome protein
GME2843_g	*MRE11*	double-strand break repair protein MRE11
GME9116_g	*MRE11*	double-strand break repair protein MRE11
GME7848_g	mutY	A/G-specific adenine glycosylase
GME100_g	*RAD5*	DNA repair protein RAD5
GME10502_g	*RAD51*	DNA repair protein RAD51
GME10948_g	*RAD55*	DNA repair protein RAD55
GME11059_g	*RAD16*	DNA repair protein RAD16
GME11219_g	*RAD50*	DNA repair protein RAD50
GME11722_g	*RAD54B*	DNA repair and recombination protein RAD54B
GME11960_g	*RAD54L, RAD54*	DNA repair and recombination protein RAD54 and RAD54-like protein
GME2608_g	*RAD52*	DNA repair and recombination protein RAD52
GME3128_g	*RAD7*	DNA repair protein RAD7
GME3142_g	*ERCC6, CSB, RAD26*	DNA excision repair protein ERCC-6
GME3893_g	*RAD16*	DNA repair protein RAD16
GME4964_g	*ERCC5, XPG*	DNA excision repair protein ERCC-5
GME5763_g	*RAD57*	DNA repair protein RAD57

GME6297_g	*RAD9*	DNA repair protein RAD9
GME6529_g	*RAD59*	DNA repair protein RAD59
**Aromatise**		
GME10351_g	*FAS1*	fatty acid synthase subunit beta, fungi type
GME12026_g	*FAS1*	fatty acid synthase subunit beta, fungi type
GME4470_g	*FAS2*	fatty acid synthase subunit alpha, fungi type
GME9105_g	*FAS2*	fatty acid synthase subunit alpha, fungi type
GME4893_g	*ATF*	alcohol O-acetyltransferase
GME9747_g	*ATF*	alcohol O-acetyltransferase
GME10382_g	*thadh*	threo-3-hydroxy-L-aspartate ammonia-lyase
GME10861_g	*AKR1A1, adh*	alcohol dehydrogenase ($NADP^+$)
GME10862_g	*AKR1A1, adh*	alcohol dehydrogenase ($NADP^+$)
GME2930_g	*AKR1A1, adh*	alcohol dehydrogenase ($NADP^+$)
GME4089_g	*AKR1A1, adh*	alcohol dehydrogenase ($NADP^+$)
GME2049_g	*frmA, ADH5, adhC*	S-(hydroxymethyl)glutathione dehydrogenase / alcohol dehydrogenase
GME6624_g	*frmA, ADH5, adhC*	S-(hydroxymethyl)glutathione dehydrogenase / alcohol dehydrogenase
GME8353_g	*frmA, ADH5, adhC*	S-(hydroxymethyl)glutathione dehydrogenase / alcohol dehydrogenase
GME2712_g	*adhP*	alcohol dehydrogenase, propanol-preferring
GME2914_g	*adhP*	alcohol dehydrogenase, propanol-preferring
GME3590_g	*adhP*	alcohol dehydrogenase, propanol-preferring
GME3869_g	*adhP*	alcohol dehydrogenase, propanol-preferring
GME6759_g	*adhP*	alcohol dehydrogenase, propanol-preferring
GME7823_g	*adhP*	alcohol dehydrogenase, propanol-preferring
GME9315_g	*adhP*	alcohol dehydrogenase, propanol-preferring
GME6375_g	*ARO1*	pentafunctional AROM polypeptide
GME9128_g	*ARO1*	pentafunctional AROM polypeptide
GME10734_g	*ARO8*	aromatic amino acid aminotransferase I / 2-aminoadipate transaminase
GME4573_g	*ARO8*	aromatic amino acid aminotransferase I / 2-aminoadipate transaminase
GME10054_g	*ARO9*	aromatic amino acid aminotransferase II
GME12739_g	*ARO9*	aromatic amino acid aminotransferase II
GME5184_g	*ARO10*	phenylpyruvate decarboxylase
GME6152_g	*ARO10*	phenylpyruvate decarboxylase
GME10810_g	*SLC7A9_15, BAT1*	solute carrier family 7 (L-type amino acid transporter), member 9/15
GME12010_g	*SLC7A9_15, BAT1*	solute carrier family 7 (L-type amino acid transporter), member 9/15
GME4791_g	*SLC7A9_15, BAT1*	solute carrier family 7 (L-type amino acid transporter), member 9/15
GME11009_g	*ALDH*	aldehyde dehydrogenase (NAD+)
GME12527_g	*ALDH*	aldehyde dehydrogenase (NAD+)
GME2736_g	*ALDH*	aldehyde dehydrogenase (NAD+)
GME2790_g	*ALDH*	aldehyde dehydrogenase (NAD+)
GME2791_g	*ALDH*	aldehyde dehydrogenase (NAD+)
GME4317_g	*ALDH*	aldehyde dehydrogenase (NAD+)
GME5805_g	*ALDH*	aldehyde dehydrogenase (NAD+)
GME5962_g	*ALDH*	aldehyde dehydrogenase (NAD+)
GME7164_g	*ALDH*	aldehyde dehydrogenase (NAD+)

GME7562_g	*ALDH*	aldehyde dehydrogenase (NAD+)
GME8402_g	*ALDH*	aldehyde dehydrogenase (NAD+)
GME8968_g	*ALDH*	aldehyde dehydrogenase (NAD+)
GME11009_g	*ALDH*	aldehyde dehydrogenase (NAD+)
GME12527_g	*ALDH*	aldehyde dehydrogenase (NAD+)
GME2736_g	*ALDH*	aldehyde dehydrogenase (NAD+)
GME2790_g	*ALDH*	aldehyde dehydrogenase (NAD+)
GME2791_g	*ALDH*	aldehyde dehydrogenase (NAD+)
GME4010_g	*E3.1.2.1, ACH1*	acetyl-CoA hydrolase
GME5207_g	*E3.1.2.1, ACH1*	acetyl-CoA hydrolase
GME2949_g	*BDH, butB*	(R,R)-butanediol dehydrogenase / meso-butanediol dehydrogenase / diacetyl reductase
GME2950_g	*BDH, butB*	(R,R)-butanediol dehydrogenase / meso-butanediol dehydrogenase / diacetyl reductase
GME9170_g	*BDH, butB*	(R,R)-butanediol dehydrogenase / meso-butanediol dehydrogenase / diacetyl reductase
GME11375_g	*DLAT, aceF, pdhC*	pyruvate dehydrogenase E2 component (dihydrolipoyllysine-residue acetyltransferase)
GME11956_g	*DLAT, aceF, pdhC*	pyruvate dehydrogenase E2 component (dihydrolipoyllysine-residue acetyltransferase)
GME4906_g	*DLAT, aceF, pdhC*	pyruvate dehydrogenase E2 component (dihydrolipoyllysine-residue acetyltransferase)
GME9977_g	*DLAT, aceF, pdhC*	pyruvate dehydrogenase E2 component (dihydrolipoyllysine-residue acetyltransferase)
GME158_g	*ERG27*	3-keto steroid reductase
GME9129_g	*ERG27*	3-keto steroid reductase
GME10852_g	*HMGCS*	hydroxymethylglutaryl-CoA synthase
GME2524_g	*HMGCS*	hydroxymethylglutaryl-CoA synthase
GME12127_g	*HMGCR*	hydroxymethylglutaryl-CoA reductase (NADPH)
GME2571_g	*HMGCR*	hydroxymethylglutaryl-CoA reductase (NADPH)
GME420_g	*HMGCR*	hydroxymethylglutaryl-CoA reductase (NADPH)
